# Engineering a 3D microfluidic culture platform for tumor-treating field application

**DOI:** 10.1038/srep26584

**Published:** 2016-05-24

**Authors:** Andrea Pavesi, Giulia Adriani, Andy Tay, Majid Ebrahimi Warkiani, Wei Hseun Yeap, Siew Cheng Wong, Roger D. Kamm

**Affiliations:** 1 Biosym IRG, Singapore–MIT Alliance for Research and Technology, 1 Create Way, 138602 Singapore, Singapore; 2 Department of Bioengineering, University of California, Los Angeles, CA 90025, USA; 3 Department of Biomedical Engineering, National University of Singapore, Singapore 117583, Singapore; 4 School of Mechanical and Manufacturing Engineering, Australian Centre for NanoMedicine, University of New South Wales, Sydney, Australia; 5 Singapore Immunology Network (SIgN), Agency for Science, Technology and Research, A*STAR, 8A Biomedical Grove, Immunos, Singapore 138648, Singapore; 6 Department of Biological Engineering, Massachusetts Institute of Technology, 77 Massachusetts Avenue, Cambridge, MA 02139-4307, USA

## Abstract

The limitations of current cancer therapies highlight the urgent need for a more effective therapeutic strategy. One promising approach uses an alternating electric field; however, the mechanisms involved in the disruption of the cancer cell cycle as well as the potential adverse effects on non-cancerous cells must be clarified. In this study, we present a novel microfluidic device with embedded electrodes that enables the application of an alternating electric field therapy to cancer cells in a 3D extracellular matrix. To demonstrate the potential of our system to aid in designing and testing new therapeutic approaches, cancer cells and cancer cell aggregates were cultured individually or co-cultured with endothelial cells. The metastatic potential of the cancer cells was reduced after electric field treatment. Moreover, the proliferation rate of the treated cancer cells was lower compared with that of the untreated cells, whereas the morphologies and proliferative capacities of the endothelial cells were not significantly affected. These results demonstrate that our novel system can be used to rapidly screen the effect of an alternating electric field on cancer and normal cells within an *in vivo*-like microenvironment with the potential to optimize treatment protocols and evaluate synergies between tumor-treating field treatment and chemotherapy.

Cancer is predicted to affect almost 17 million people worldwide by 2020^1^,^2^. To reduce the cancer burden, various research efforts have been made to improve both diagnostic and therapeutic strategies. The most common types of cancer therapy clinically rely on radiation, chemotherapy, and surgical resection. Despite advances in the respective technologies and novel therapeutic strategies under investigation, an optimal treatment with reduced risks and side effects has yet to be discovered. Therefore, a strong incentive exists to explore alternative forms of therapy. One approach that has recently been studied is the use of electromagnetic and radiofrequency fields as a complement to current care regimes^3^,^4^.

The roots of electromagnetic cancer treatment can be traced back to the 1920s when Lakhovsky developed the “Radio-Cellular-Oscillator” to apply radio frequencies to cancer cells^5^. In the 1980–1990s, The National Council on Radiation Protection and Measurements recommended a reduction in the allowable exposure intensity limits for electromagnetic radiation, reducing the level of interest in this treatment modality^6^. However, because of the persistent limitations of current cancer therapies, interest in alternative forms of treatment including electromagnetic stimulation, have reemerged. The different electromagnetic stimulation methods can be distinguished by their wave frequency, voltage intensity, and duration of application as well as the mechanism by which they affect biological tissues. For instance, low frequencies (<1 kHz) are typically used to stimulate excitable tissues (nerve, muscle, heart), whereas frequencies higher than 10 MHz, which are often used for tumor ablation^7^, cause tissue heating. Electromagnetic stimulation has recently showed promising results in gene delivery strategies for various applications ranging from wound healing to cancer treatment^8^. DNA, cytokines, or drugs can be introduced into cells by transferring nanosecond pulsed electric fields to permeabilize cell membranes as an extension of the more classical electroporation. Palti *et al*. coined the term ‘tumor-treating fields’ (TTFs)^9^ after showing that low-intensity (1–2 V/cm), intermediate frequency (100–200 kHz) alternating electric fields inhibit actively dividing cells by disrupting mitosis^10^,^11^. These researchers proposed that during cellular processes such as mitosis, polar molecules (including the microtubule spindle) generate intracellular electric fields via motion synchronization^12^, making cells responsive to applied electric fields. Alternating electric fields can cause misalignment of the tubulin subunits, preventing polymerization of mitotic spindles. In addition, finite element modeling has demonstrated that electrical energy can affect cytokinesis by disrupting the mitotic furrow via dielectrophoretic effects^7^. The optimal inhibitory effects of an alternating electric field on cancer cells are frequency and intensity dependent and have been observed to be inversely related to cell size^13^. Encouraged by these results, two companies, NovoCure and TheraBionic, have been launched based on the TTF technology. Recent advances include favorable results of NovoTTF-100A in a phase III clinical trial for recurrent glioblastoma over chemotherapy^14^ and the positive synergistic effects of TTF with the drug Pemetrexed (Eli Lilly) for non-small-cell lung cancer treatment^15^,^16^.

Despite promising *in vitro* and *in vivo* results, and even after U.S. Food and Drug Administration (FDA) approval, the response to TTF treatment remains cautious, possibly because of the lack of an *in vitro* 3D platform to efficiently test the therapy and aid a scientific understanding of the precise mechanisms that lead to inhibition of key cancer stages such as growth, angiogenesis, and metastasis. It is crucial in fact to further investigate the signaling mechanisms, apoptotic pathways, and any possible adverse consequences that previous studies have not yet discovered. In addition, further studies are needed to identify parameter ranges that are optimized to limit cancer cell proliferation while minimizing detrimental effects to other normal neighboring cells present in *in vivo* tissues.

For this purpose, we engineered a microfluidic device with embedded contactless electrodes to stimulate cells with an alternating electric field in a highly controlled microenvironment. This system is the first microfluidic system in the literature to offer the opportunity to investigate the effects of TTF on cancer cells in a 3D extracellular-matrix-like hydrogel. Cancer cells were cultured alone (mono-culture) or jointly with non-cancerous cells (co-culture). Moreover, the platform offers other well-known advantages of microfluidics technology such as reduced volume of reagents and lower costs, portability, and the capability of observing phenomena in a 3D culture condition in real time, which better mimics the *in vivo* configuration compared with classical culture dishes^17^.

## Methods

### Fabrication of microfluidic device with injectable electrodes

**Figure 1:**
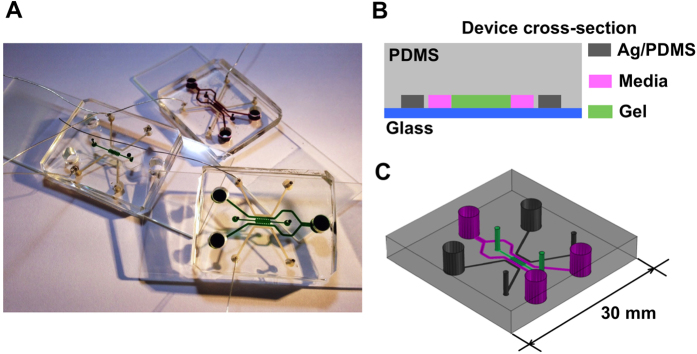
Microfluidic device for TTF application. (**A**) Photo of three devices filled with food color dyes. Device cross section (**B**) and 3D CAD overview (**C**) with the conductive mixture (Ag/PDMS) in dark grey, cell culture media in pink, and 3D hydrogel region in green.

The device design consisted of two layers: a polydimethylsiloxane (PDMS) layer and a glass coverslip substrate. The PDMS layer was fabricated using replica molding (soft lithography) of a master mold created with a 120-μm-thick layer of SU-8 photoresist (Microchem Corporation, Newton, MA, USA) patterned on a silicon wafer by photolithography. The PDMS was prepared by mixing a pre-polymer base and a cross-linker (Sylgard 184 Silicone elastomer kit, Dow Corning, Midland, MI, USA) at a 10:1 weight ratio. After vacuum degassing, the mixture was poured onto the master mold and cured for 2 h at 80 °C. The PDMS layer was gently separated from the master mold and trimmed. Access holes of different diameters (Fig. 1; 1 mm for the gel channel, 4 mm for the fluidic channels, and 4 or 2 mm for the PDMS–silver electrode channels) were created by punching through the PDMS with Uni-Core^TM^ punchers (Sigma-Aldrich, St. Louis, MO, USA). After autoclave sterilization and oxygen plasma treatment, each PDMS piece was bonded to a glass coverslip. The assembled device (Fig. 1) consisted of 5 channels: one 1.3-mm-wide central 3D gel region (green in Fig. 1) communicating with two lateral 500-μm-wide fluidic channels for the cell culture medium (pink in Fig. 1), and two more-external 500-μm-wide side channels to inject the conductive mixture (grey in Fig. 1), separated from the medium channels by a 150-μm-wide PDMS wall. The microfluidic channels dedicated for the electrodes were easily fillable with injectable conductive mixtures similarly to our previous work^18^,^19^. Specifically, a new conductive mixture was prepared consisting of 10-μm silver flakes (Sigma–Aldrich) mixed with PDMS pre-polymer at a 4:1 weight ratio and injected with a syringe via the 4-mm access hole; a conductive wire was inserted into the output (2-mm hole) before curing the pre-polymer to create a closed electrical circuit. The injected electrodes were placed on a hotplate for 4 h at 120 °C. The electrode conductivity in each device was tested, and using the silver flake-PDMS mixture lead to a significant reduction in resistance to a few ohms with respect to our previous systems^18^,^19^. Before the cell culture, the device channels were coated with 1 mg/ml of poly-D-lysine (Sigma–Aldrich) for 2 h, washed with sterile water, and dried at 80 °C for at least 24 h to restore hydrophobicity.

### Cell culture and cancer aggregate formation

Two cancer cell lines were used: 1) human breast adenocarcinoma cell line (MDA-MB-231) and 2) an mCherry transfected human lung adenocarcinoma cell line (A549), both cultured in high-glucose Dulbecco’s modified Eagle’s medium (DMEM) (Life Technologies, Carlsbad, CA, USA) supplemented with 10% fetal bovine serum (FBS) (Life Technologies) and 1% penicillin-streptomicin (Life Technologies). Human umbilical vein endothelial cells (HUVECs, Lonza, Basel, Switzerland) were cultured in endothelial growth medium (EGM-2, Lonza), and cells at passages 5 to 7 were used for the experiments. The cell cultures were maintained in a humidified incubator at 37 °C and 5% CO_2_ and grown to 80% confluence before passage. A549 aggregates were generated in a laser-patterned custom-made Petri dish, as previously described^20^. The aggregates were retrieved after 4 days and sieved sequentially through 100- and 40-μm-diameter cell strainers to yield aggregates 40–100 μm in diameter, which were then enriched by centrifugation.

### Cell seeding into the microfluidic device

A collagen type I solution (2.5 mg/ml) was prepared as described previously^21^. For the individual cell seeding, MDA-MB-231 cells were mixed with the collagen solution at a concentration of 3 × 10^5^ cells/ml, and 8 μl of the solution was injected into the gel region of each device. For the aggregate seeding, A549 cells were preferred to MDA-MB-231 as they readily form aggregates in 4 days. The A549 aggregates were mixed with the collagen solution and injected into the devices and then incubated at 37 °C for 30 min to allow gel polymerization before filling the fluidic channels with cell culture medium. The A549 aggregates were mixed with the collagen solution and injected into the gel region of the devices and then incubated at 37 °C for 30 min to allow gel polymerization before filling the fluidic channels with cell culture medium. For the co-culture of cancer cells (either MDA-MB-231 or A549) with HUVECs, the fluidic channels were coated with 50 μg/ml of fibronectin solution (Sigma-Aldrich) for 45 min before each experiment, and the HUVECs were injected into the fluidic channels with the medium at a concentration of 3 × 10^6^ cells/ml. When all the channels were filled, the microfluidic platforms were incubated at 37 °C and 5% CO_2_ for 4 h to ensure cell adhesion before starting the stimulation by connecting the devices to the frequency generator. All the experiments were performed in static (no-flow) culture conditions and the culture media was changed daily from the inlet/outlet ports.

### Electric field stimulation

Solutions of suspended MDA-MB-231 cells and aggregates composed of A549 cells, mono-cultured or co-cultured with HUVECs, were subjected to alternating electric fields at frequencies of 150 and 200 kHz, respectively, for 3 days at an intensity of 1.1 V/cm, following the *in vivo* and *in vitro* stimulation parameters proposed by^9^,^10^ and used as the golden standard. A positive control was implemented in the experimental design by adding 10% DMSO in the culture media to confirm positive staining in case of cell death (DMSO dose tested Supplementary Figure S1).

### Cell staining and microscopy imaging

To detect and visualize MDA-MB-231 breast cancer cells in the apoptotic cycle in real time, CellEvent^®^ Caspase-3/7 Green Detection Reagent (Life Technologies) was used following the manufacturer’s protocol. For A549 lung cancer cell aggregates, the nuclei were labeled with NucBlue for live staining (Life Technologies), and cell impermeable nuclear dye DRAQ7 (Biolegend, San Diego, CA, USA) was added in the culture medium at a concentration of 3 μM to discriminate between live and dead cells. ActinGreen 488 ReadyProbes Reagent (Life Technologies) was used to stain for F-actin filaments (Representative images of the devices at 72 h showed in Supplementary Figure S2). Images were captured using a LSM-780 Zeiss confocal microscope (Zeiss, Oberkochen, Germany).

### Immunocytochemistry

Cells in the device were fixed with 4% formaldehyde in PBS for 15 min, washed twice in 1xPBS and stored at 4 °C. For the immunostaining, cells were permeabilized for 15 min in 0.5% Triton X-100, washed twice in 1xPBS and incubated for 3 h with a blocking solution containing 3%BSA and 5% goat serum in PBS. Rabbit polyclonal antibody against Ki-67 (Abcam, Cambridge, UK) and chicken polyclonal antibody against MMP-14 (Sigma) were diluted 1:200 in PBS containing 0.5%BSA and added to the respective devices. As a control, one device was incubated in PBS containing 0.5%BSA without the primary antibodies. After overnight incubation at 4 °C and 5 washing steps in 1xPBS, an Alexa Flour 647 anti-rabbit and a Alexa Fluor 568 anti-chicken secondary antibodies (Life Technologies) were diluted 1:200 in PBS containing 0.5%BSA and NucBlue, and added in the respective devices. After 2 h, all the devices were washed 5 times in 1xPBS and imaged.

### Enzyme-Linked Immunosorbent Assays

To investigate the secretion of inflammatory markers upon the electric field stimulation, supernatant collected from the devices containing HUVECs alone were examined by ELISA using DuoSet ELISA development kits (R&D Systems) against human Interleukin-6 (IL-6) and human Interleukin-8 (IL-8). Supernatants from at least 3 devices for each condition were collected. Assays were performed according to the manufacturer’s instructions and run in duplicates.

### Image analysis and statistics

The acquired images were visualized and analyzed with the image-processing package Fiji (Image J software) and IMARIS (Bitplane Scientific software). The cell density was calculated by counting the number of cells in each randomly selected region of interest (ROI) and dividing it by the ROI volume to obtain a quantification independent from the considered volume. The percentage of caspase-activated nuclei was calculated using the ratio between the number of cells expressing green fluorescence signal and the total number of cells in each ROI. The normalized cancer aggregate dispersion was calculated as described before^22^ and reported here for clarity. The spatial coordinates of the centroids of all the cell nuclei forming the cancer aggregate were determined using IMARIS and imported in Matlab (MathWorks, Natik, MA, USA) to calculate the aggregate dispersion^22^,^23^. For *N* nuclei in a given spheroid, the geometric centers of the nuclei are represented by (*x*_*i*_, *y*_*i*_, *z*_*i*_), *i* = 1, 2, …*N*. The aggregate center is then represented as


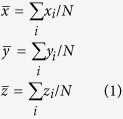


From these, the standard deviation can be calculated as


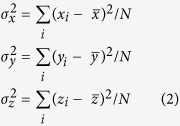


and the normalized dispersion can be represented as


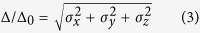


where the normalizing value (Δ_0_) is the dispersion value at *t* = 0. All the data were calculated considering at least three devices and three ROIs for each device. Unless otherwise stated, the data were plotted as mean ± standard error of the mean (SEM) using Prism (GraphPad software), and statistical significance was determined using a two-tailed unpaired Student’s t-test.

### Electric field model

**Figure 2:**
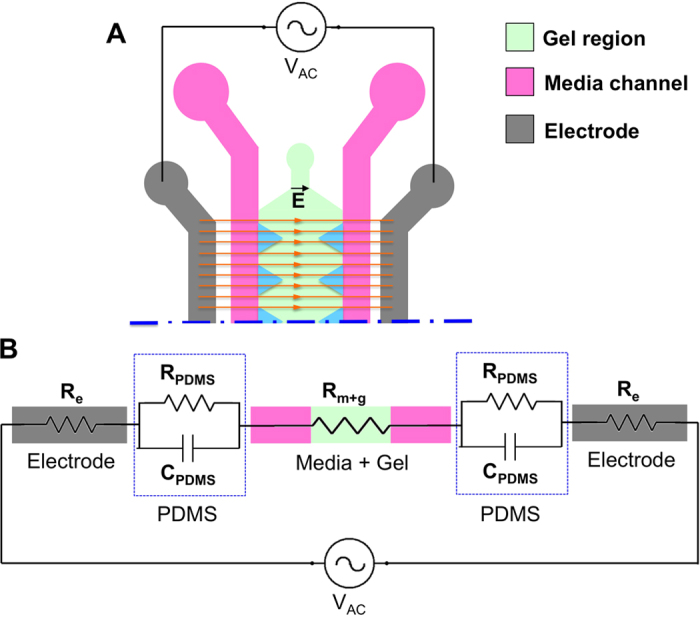
Equivalent electric circuit model of the microfluidic device. (**A**) Scheme of the device channels that constitute the circuit. (**B**) Equivalent electric circuit. The electrodes (in gray) are modeled as resistor elements (R_e_). Each PDMS element is modeled as a resistor (R_pdms_) in parallel with a capacitor (C_pdms_). The cell culture media (in pink) and the gel region (in green) are modeled as resistor elements (R_m+g_). The frequency generator is connected to the electrodes to close the circuit.

To estimate the voltage across the collagen gel region where cancer cells were seeded, the microfluidic device was modeled with an equivalent electrical circuit^24^ (Fig. 2). The PDMS layer was modeled as a resistor–capacitor (RC) pair in parallel and in series, with the other components of the microfluidic device modeled as resistors (Fig. 2). The values of the used parameters are summarized in Table 1 and in Supplementary Table 1.

The impedance of PDMS (Z_PDMS_), which was modeled as an RC pair, was calculated as Z_PDMS_ = 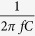, where *f* is the frequency and *C* is the capacitance. The relationship between the voltage across the collagen gel region (*V*_*gel*_) and the input voltage (*V*_*in*_) was obtained by solving the equivalent circuit as


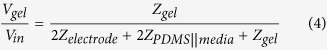


By considering the dimensions of the microfluidic device and target frequencies, the required input voltage was calculated to provide the desired voltage across the collagen gel region. Details of the calculation are provided in the Supplementary Information.

## Results

The TTF device, consisting of five parallel microfluidic channels as described in the Materials and Methods section, allows for the first time the application of controlled electrical field stimulation (frequency, amplitude, and waveform) to cancer cells in a 3D hydrogel mimicking the extracellular matrix.

### Electric field stimulation inhibits proliferation of breast cancer cells in mono-culture

**Figure 3:**
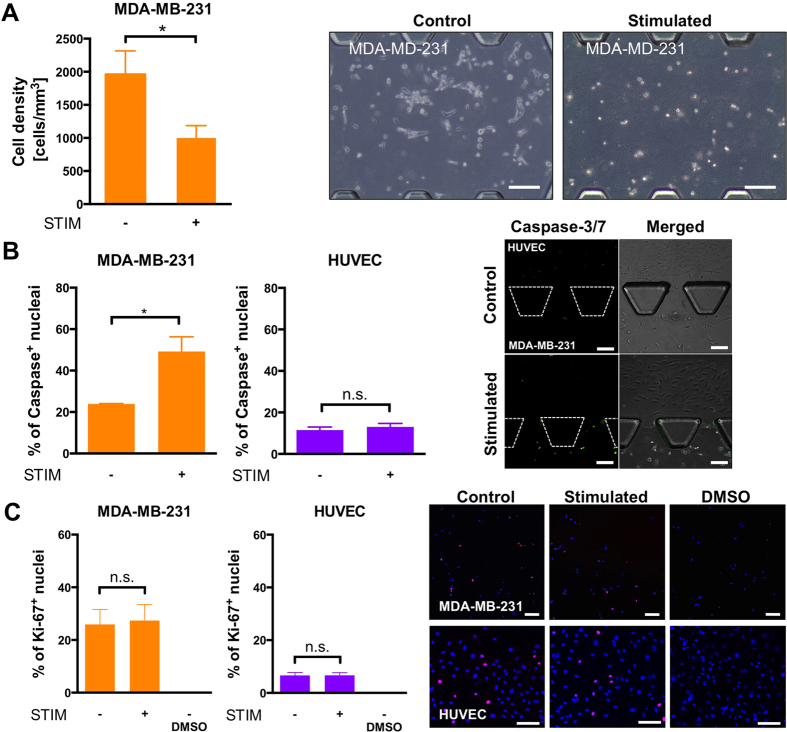
Effects of alternating electric field on breast cancer cells in monoculture or in co-culture with endothelial cells. (**A**) Cell density and morphology of breast cancer cells (MDA-MB-231) stimulated at a frequency of 150 kHz and intensity of 1.1 V/cm for 3 days. (**B**) Percentage of caspase dye activation in breast cancer cells (MDA-MB-231) and endothelial cells (HUVEC) stimulated at a frequency of 150 kHz and intensity of 1.1 V/cm. (**C**) Percentage of Ki-67^+^ breast cancer cells (MDA-MB-231) and endothelial cells (HUVEC) stimulated at a frequency of 150 kHz and intensity of 1.1 V/cm. Representative images with Ki-67 expression (in red) and blue nuclei. Unpaired t-test, *p < 0.05, n.s = not significant. Scale bars = 100 μm.

As a proof-of-concept of device capabilities, MDA-MB-231 cells were homogenously dispersed in collagen gel to model the extracellular environment, injected into the microfluidic devices, and subjected to an alternating electric field of 150 kHz at an intensity of 1.1 V/cm for 3 days. For unstimulated MDA-MB-231 cells, the mean cell density count was ~2000 cells/mm^3^, whereas under electrical stimulation, the count was ~1000 cells/mm^3^. In addition to this reduction in cell density, when the MDA-MB-231 cells, embedded in the 3D matrix, were exposed to electric field stimulation, they exhibited rounded morphologies quite distinct from the mesenchymal morphologies of the unstimulated ones (Fig. 3A).

### Proliferation of breast cancer cells but not endothelial cells is reduced in co-culture

MDA-MB-231 cells were homogenously dispersed in collagen gel as in the mono-culture experiments previously described and co-cultured in the device with HUVECs seeded in the lateral fluidic channels. The rationale behind adding the endothelial cells is to have an *in vitro* model with a non-neoplastic tissue (HUVEC) representing a vessel in co-culture and communicating with the cancer cells in 3D.

The effect of TTF on the proliferation of MDA-MB-231 breast cancer cells was quantified as a percentage of Ki-67 positive cells (Fig. 3C). Similar Ki-67 expression in breast cancer cells was observed in the stimulated devices relative to the control condition (Fig. 3C). In particular, the mean percentages of Ki-67^+^ cells ± SEM at 72h were 27.37 ± 6.12% and 25.94 ± 5.61% for the stimulated and control conditions, respectively. Similarly, proliferation of HUVECs was comparable for both the stimulated and unstimulated conditions (Fig. 3C). Specifically, the percentages of Ki-67^+^ cells ± SEM at 72h was 6.69 ± 1% for the stimulated endothelial cells and 6.63 ± 1.08% without stimulation.

Apoptosis of the HUVECs remained low and constant at 72 h experimental time for both the stimulated and unstimulated conditions (Fig. 3B). Specifically, caspase dye activation in HUVECs under electrical stimulation was observed in 13.04 ± 1.67% (mean ± SEM) of the HUVEC population; without stimulation, the value was 11.47 ± 1.46% of the HUVEC population (the total HUVEC population was quantified using nuclei staining). Although we observed a slight increase in the percentage of apoptotic HUVEC cells, the difference was not statistically significant. However, caspase activation was doubled in MDA-MB-231 cells under electric field stimulation (49.20 ± 7.1%) as compared with those without stimulation (23.88 ± 0.22%).

MMP-14 expression of MDA-MB-231 and HUVEC cells was investigated by quantification of the percentage of MMP-14 positive cells. MMP-14 was not expressed on the MDA-MB-231 cells while HUVECs presented similar MMP-14 expression in the stimulated and control devices (Supplementary Figure S3A).

### Electric field stimulation inhibits proliferation and dispersion of lung cancer cell aggregates

To further validate the device capabilities and investigate the electric field effect applied to a 3D cancer mass, we cultured A549 lung carcinoma cells to form aggregates. Co-culture with HUVECs promoted A549 carcinoma aggregate dispersion as demonstrated in previous studies using a similar system^22^,^23^; therefore, we decided to include a HUVEC monolayer in the device to better mimic the *in vivo* microenvironment.

**Figure 4:**
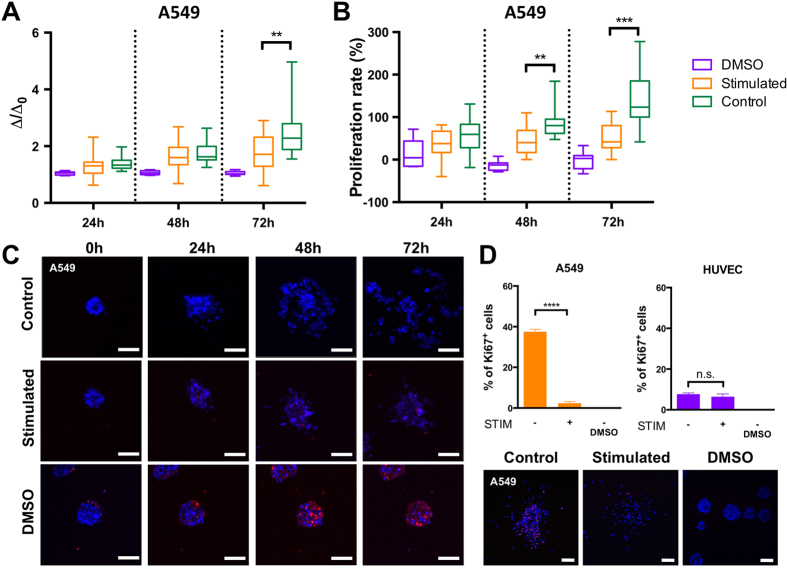
Effects of alternating electric field on lung cancer cell aggregates. Normalized dispersion (Δ/Δ_0_) (**A**) and proliferation rate (**B**) of A549 lung cancer cell aggregates after 24, 48, and 72 h of stimulation at a frequency of 200 kHz and 1.1 V/cm intensity. (**C**) Representative confocal images of A549 aggregates at 0, 24, 48, and 72 h under DMSO treatment (positive control), under electric field stimulation and without stimulation (negative control). Blue = cell nuclei and red = dead cells. Scale bars = 50 μm. (**D**) Percentage of Ki-67^+^ lung cancer cells (A549) and endothelial cells (HUVEC) stimulated at a frequency of 200 kHz and intensity of 1.1 V/cm. Representative images with Ki-67 expression (in red, lower panel). Unpaired t-test, **p < 0.01, ***p < 0.001, ****p < 0.0001, n.s = not significant. Scale bars = 100 μm.

Alternating electric field at a frequency of 200 kHz and intensity of 1.1 V/cm was applied to the A549 aggregates seeded in collagen gel, and confocal images were then taken every 24 h for 3 days. The normalized dispersion and proliferation rate of carcinoma aggregates at 24, 48, and 72 h were computed, revealing a progressive increase in dispersion in both stimulated and control conditions with no dispersion observed for the aggregates with DMSO (Fig. 4A,C). However, at 72 h, carcinoma aggregates dispersed significantly more (p < 0.01) when not subjected to the electric field stimulation (control condition) (Fig. 4A,C). Therefore, after 72 h of continuous stimulation, the electric field effectively reduced carcinoma cell aggregate dispersion. MMP-14 expression of A549 and HUVEC cells was investigated by quantification of the percentage of MMP-14 positive cells. Both the A549 and the HUVECs presented similar MMP-14 expression in the stimulated and control devices although MMP-14 expression of A549 was higher compared to HUVECs (Supplementary Figure S3B).

The proliferation rate of cancer cells within the aggregates, defined as the percentage increase in the number of cells relative to 0 h, was observed to be significantly lower under TTF at 48 h relative to the control condition, with the difference increasing at 72 h (Fig. 4B). In particular, the mean proliferation rates ± SEM at 48 h were 46.83 ± 9.32% and 88.15 ± 10.54% for the stimulated and control conditions, respectively. At 72 h, the proliferation rates were 50.77 ± 8.55% and 142.10 ± 18.90% for the stimulated and control conditions, respectively (Fig. 4B). Therefore, the electric field reduced the average proliferation rate by roughly 2-fold after 48 h and almost 3-fold after 72 h.

The effect of TTF on proliferation of A549 lung cancer cells within the aggregates was quantified as a percentage of Ki-67 positive cells (Fig. 4A). A clear decrease in Ki-67 expression for lung cancer cells was observed in stimulated devices relative to the control condition (Fig. 4A). In particular, the mean percentages of Ki-67^+^ cells ± SEM at 72h were 2.38 ± 0.77% and 37.54 ± 1.19% for the stimulated and control conditions, respectively. In contrast, proliferation of the HUVECs was similar for both the stimulated and unstimulated conditions (Fig. 4A). Specifically, the % of Ki-67^+^ cells ± SEM at 72h was 6.40 ± 1.39% for the stimulated endothelial cells and 7.65 ± 0.61% without stimulation.

### Secretion of IL-6 and IL-8 by HUVECs does not change after 72h of electric field stimulation

**Figure 5:**
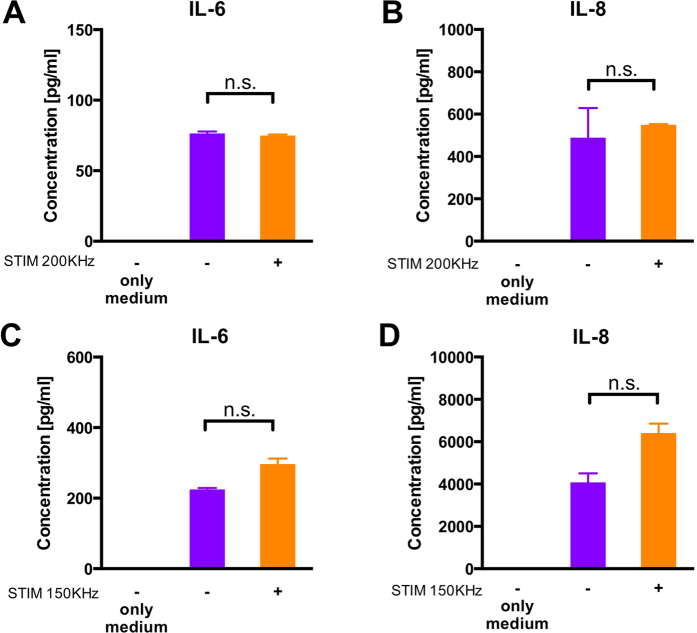
Expression levels of IL-6 and IL-8 in the cell supernatants by Enzyme Linked Immunosorbent Assays (ELISA). Concentrations of IL-6 (**A,C**) and IL-8 (**B,D**) secreted from HUVECs cultured in the microfluidic device either not stimulated (−, in violet) or stimulated (+, in orange) at 200 KHz (**A,B**) or 150 KHz (**C,D**). Supernatants from at least 3 devices for each condition were collected. Statistical analysis was performed by a t-test with Welch’s correction, n.s = not significant.

To study the possible inflammation of HUVECs due to the TTF stimulation, soluble expression of IL-6 and IL-8 in the supernatants collected from the microfluidic devices were assessed by ELISA. The results revealed that after 3 days of stimulation at 200 KHz, HUVECs secreted inflammatory cytokines IL-6 and IL-8 at similar levels compared to the unstimulated condition (Fig. 5A,B). Similar results were obtained after 3 days of HUVEC stimulation at 150 KHz where a slight increase in soluble expression levels of IL-6 and IL-8 was observed that was not significantly different from the unstimulated condition (Fig. 5B,C).

## Discussion

The use of alternating electric fields has shown potential benefits in cancer treatment either in combination with more traditional forms of treatment or as a stand-alone method for cancers resistant to existing therapies^3^,^4^. To date, however, it has been difficult to identify optimal protocols, and the potential side effects on non-cancerous cells are relatively unexplored. Indeed, very few works are available in the literature on the effect of electric fields on tissues cultured *in vitro*^7^,^9^,^25^ and specifically on the selective properties of a TTF in affecting cancer cell fate while leaving undisturbed non-carcinogenic cells for a co-culture condition. Previous *in vitro* studies have provided helpful insights such as the alternating electric fields disrupting mitotic spindle synchronization. However, these experiments that have largely been performed in 2D standard culture assays and in mono-culture condition^9^ may not fully capture the 3D characteristics of physiological cancer cell behavior such as cell migration and dispersion.

The *in vitro* microfluidic device presented here is the first microfluidic platform in the literature able to apply an electric field and represents the first *in vitro* system considering TTF applied to cells in 3D. Moreover, for the first time, this microfluidic system allows the study of TTF effects on malignant and non-malignant cells in co-culture condition. Importantly, this device represents a novel technical development compared with our previous devices. Namely, while the device developed in Pavesi *et al*.^19^ applies electric and mechanical stimulation to cells cultured in the device, the present device treats cells with an electric field without any electric current passing through the cells. The stimulation is performed using newly conceived silver-PDMS electrodes incorporated into the device.

Therefore, our device may address previous limitations by facilitating the application and exploration of TTF technology to different cells in a controlled 3D microenvironment. A major advantage of our microfluidic platform is the compatibility with confocal microscopy techniques to image spatially defined 3D co-culture systems while monitoring the cell viability and cancer invasiveness of multiple cell types subjected to the same TTF. Must be noticed that the central hydrogel region is separated from the fluidic channels by posts that allow co-cultured cells’ communication trough paracrine signaling. Additionally, the system enables collection of the cell supernatant for cytokine analysis.

Our experimental platform can be utilized to obtain further insights into the mechanisms of electric field stimulation on cancer cells in 3D as well as the specific parameter values that affect tumor growth but do not harm surrounding, non-cancerous cells present in the tissue such as endothelial cells. Capillary network lined by vascular endothelial cells, in fact, plays an essential role in tumor progression either at primary site through angiogenesis and following intravasation of cancer cells in circulation or at secondary sites, where circulating tumor cells cross a blood vessel to proliferate in a different tissue^26^,^27^. One of the main function of blood vessels lined by endothelial cells is to provide nutrients to tumor cells that, together with tumor-associated macrophages (TAMs), stimulate new vascular network formation through secretion of growth factors such as vascular endothelial growth factor (VEGF)^28^. Furthermore, endothelial cells express adhesion molecules to sustain the physiological adhesion of leukocytes but circulating tumor cells also interact with these adhesion molecules as selectins or vascular cell-adhesion molecule-1 (VCAM-1) on endothelial cells to extravasate^29^. Regarding more specific interactions between endothelial cells and the solid tumors investigated in this work, lung adenocarcinoma cells in the presence of endothelial cells were found to enhance their dispersion through epithelial-mesenchymal-transition (EMT)^22^,^23^ and to increase their migration via VEGF and PDGF-BB secretion^30^.

Interestingly, endothelial cells were also showed to play a tumor-inhibitory effect on lung cancer growth by Dll4/Notch1/PTEN signaling pathway^31^. Breast cancer cells were, similarly, found to enhance adhesion and invasion properties through a dynamic interplay with endothelial cells^32^. Given the essential role of blood vessels in tumor progression, by including endothelial cells in the TTF device, we provide not only a way to test the electric field treatment on non-malignant cells but also a more physiologically relevant microenvironment. Therefore, we have demonstrated the novel capability of the system in applying alternating electric field stimulation and directly observing its effects on representative single cancer cells (MDA-MB-231) or cell aggregates (A549), cultured individually or co-cultured with endothelial cells (HUVECs). We purposely selected a frequency already tested by other groups, considered as the golden standard, in a mono-culture or *in vivo* configurations^9^,^10^ and implemented them into our 3D co-culture model to evaluate the cell response.

The unstimulated breast cancer cells exhibited a more rounded, as opposed to mesenchymal, morphology that is often indicative of a reduced cell motility and metastatic invasive potential in line with previous publications^10^. Most notably, a 50% decrease in cell density was observed in the stimulated breast cancer cells compared with unstimulated ones.

In co-culture experiments of breast cancer with endothelial cells, apoptosis in breast cancer cells doubled after electric field stimulation, whereas apoptosis of endothelial cells was not significantly affected, indicating that the apoptotic effects of the alternating electric field was limited to the breast cancer cells at the applied frequency and intensity. Furthermore, the secretion of inflammatory cytokines (IL-6 and IL-8) by endothelial cells was not significantly affected after electric field stimulation.

Our results support the hypothesis that the mechanism for inhibition of cellular activities using alternating electric field is cell-type dependent.

Note that the HUVEC doubling time (DT) according to the vendor is ~15–48 h, which is consistent with the value of ~27 h observed in the literature^33^, whereas for the MDA-MB-231 cancer cells, the DT ranges between ~28 h^34^ and ~38 h (ATCC), thus, both cell types should have been actively dividing and been affected for treatment over 72 h. However, the versatility of our TTF device indeed offers the possibility of varying the treatment time to address the difference in cell cycles.

For the stimulation experiments with cancer aggregates (mimicking a tumor mass), the metastatic potential of lung carcinoma cells, quantified in terms of cancer aggregate dispersion, was significantly reduced under TTF stimulation. Furthermore, the average proliferation rate of lung cancer cells resulted in an almost three-fold reduction for the stimulated condition compared with the control without stimulation. The inhibition of the proliferative capacity of lung cancer cells was verified by a dramatic reduction of Ki-67^+^ cells. Although the ultimate goal of cancer therapy must be the complete eradication of the malignant cells, the possibility of extending life by controlling cancer cell growth and by preventing metastatic dissemination also has enormous value.

## Conclusions

A novel microfluidic device was designed, fabricated, and used to apply electric field stimulation (TTC) to different cell types cultured in 3D matrices. This original approach can provide new insights, not only into the understanding of the treatment effects on cancer cells but also on healthy tissues, allowing the possible side effects of each treatment to be estimated. Moreover, the described 3D microfluidic platform may facilitate further investigation into the usefulness of alternating electric field stimulation in cancer treatment or into the synergy of alternating electric field stimulation in combination with other, more conventional therapeutic approaches. The presence of fluidic channels flanking the 3D cell culture region, in fact, may easily allow the combination of TTF with chemotherapy to optimize the treatment for each cancer and eventually to tune patient-specific therapeutic options for personalized medicine.

## Additional Information

**How to cite this article**: Pavesi, A. *et al*. Engineering a 3D microfluidic culture platform for tumor-treating field application. *Sci. Rep.* **6**, 26584; doi: 10.1038/srep26584 (2016).

## Supplementary Material

Supplementary Information

## Figures and Tables

**Table 1:** Parameters for the estimation of the input voltage.

Parameters	Values	Units	Ref.
Resistivity, electrode/silver-PDMS (ρ_electrode_)	1.67 × 10^−5^	Ω·m	35
Resistivity, media (ρ_media_)	0.67	Ω·m	36
Resistivity, collagen gel (ρ_gel_)	800	Ω·m	37
Resistivity, PDMS (ρ_PDMS_)	1.2 × 10^12^	Ω·m	36
