# Supplementary material for: Engineering a 3D microfluidic culture platform for tumor-treating field application
Source: Sci Rep. 2016 May 24;6:26584. doi: 10.1038/srep26584 (PMC4877588; doi:10.1038/srep26584)
Supplement: Supplementary Information [file srep26584-s1.pdf]

Engineering a 3D microfluidic culture platform for tumor-treating field application

Andrea Pavesi[a,*], Giulia Adriani[a,*], Andy Tay[a,*], Majid Ebrahimi Warkiani[a] , Wei Hseun Yeap[c], Siew Cheng Wong[c] and Roger D. Kamm[a,b]

[a]Biosym IRG, Singapore–MIT Alliance for Research and Technology, 1 Create Way, 138602 Singapore
[b]Department of Biological Engineering, Massachusetts Institute of Technology, 77 Massachusetts Avenue, Cambridge, MA 02139-4307, USA
[c]Singapore Immunology Network (SIgN), Biomedical Sciences Institute, A*STAR, 8A Biomedical Grove, Immunos, Singapore 138648

*Equal contribution

SUPPLEMENTARY INFORMATION

Electric field model

All the numerical parameters used in the model are summarized in the following table S1.

Table s1: Numerical values used in the model

	**Value**	**Unit**	**Cit.**
**Material propriety**			
PDMS relative permittivity (ε_r^{PDMS})	2.75	*[-]*	1
PDMS resistivity (ρ_{PDMS})	$1.2 * 10^{12}$	*Ohm * m*	2
Culture media resistivity (ρ_{media})	0.67	*Ohm * m*	2
Collagen gel resistivity (ρ_{gel})	800	*Ohm *m*	3
Ag-PDMS electrode resistivity ($\rho_{electrode}$)	$1.67*10^{-5}$	*Ohm *m*	4
**Geometry**			
Electrode width ($w_{electrode}$)	5*10-4	*m*	
PDMS channel width (w_{PDMS})	1*10-4	*m*	
Culture media channel width (w_{media})	5*10-4	*m*	
Gel channel width (w_{gel})	1.3*10-3	*m*	
Height of the channels ($h_{channel}$)	1.2*10-4	*m*	
Length of the device channel ($l_{channel}$)	3.2*10-3	*m*	
**Constants**			

Permittivity in vacuum (ε_0)	8.85*10-12	*F/m*
**Adopted stimulation frequencies**		
MDA-MB-231	150	*KHz*
A549	200	*KHz*

Calculations:

The calculations were used to identify the stimulation amplitude to apply at the electrodes for the different stimulation frequencies.

To determine the voltage at the electrodes, the following relation was used:

$$V_{IN} = V_{gel} \cdot \frac{Z_{tot}}{Z_{gel}}.$$

The total impedance of the electrical equivalent circuit represented in Fig. 2 was calculated as

$$Z_{tot} = 2 \cdot R_{electrode} + R_{gel} + 2 \cdot R_{media} + 2 \cdot \frac{X_{PDMS}^2 R_{PDMS} - i X_{PDMS} R_{PDMS}^2}{R_{PDMS}^2 + X_{PDMS}^2} \; [\Omega],$$

where the resistance associated with the silver–PDMS electrodes was calculated using the following relation:

$$R_{electrode} = \frac{\rho_{electroce} \cdot w_{electrode}}{l_{channel} \cdot h_{channel}}.$$

The resistance associated with the PDMS was estimated using the following relation:

$$R_{PDMS} = \frac{\rho_{PDMS} \cdot w_{PDMS}}{l_{channel} \cdot h_{channel}}.$$

The reactance of the PDMS was proportional to the applied frequency of stimulation:

$$X_{PDMS} = -\frac{1}{\omega C} = -\frac{1}{2\pi f C_{PDMS}},$$

where f is the frequency used for the stimulation and C is the PDMS capacitance, calculated as

$$C_{PDMS} = \varepsilon_0 \cdot \varepsilon_r^{PDMS} \cdot \frac{l_{channel} \cdot h_{channel}}{w_{PDMS}}.$$

The resistances associated with the median and gel were calculated as

$$R_{media} = \frac{\rho_{media} \cdot w_{media}}{l_{channel} \cdot h_{channel}}$$

$$R_{gel} = \frac{\rho_{gel} \cdot w_{gel}}{l_{channel} \cdot h_{channel}}.$$

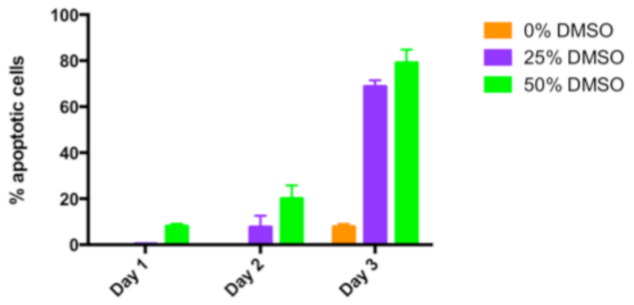

Supplementary Figure S1. Percentage of apoptotic breast cancer cells (MDA-MB-231) tested as positive control for different concentrations of DMSO diluted in the culture media.

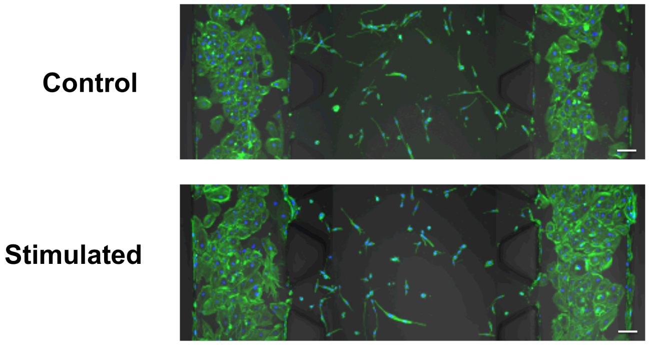

Supplementary Figure S2. Representative figures of the whole microfluidic device section with endothelial cells co-cultured with breast cancer cells embedded in 3D collagen type I hydrogel. Images show the devices at 72h, either stimulated or non-stimulated (control). Scale bars = 100 μm.

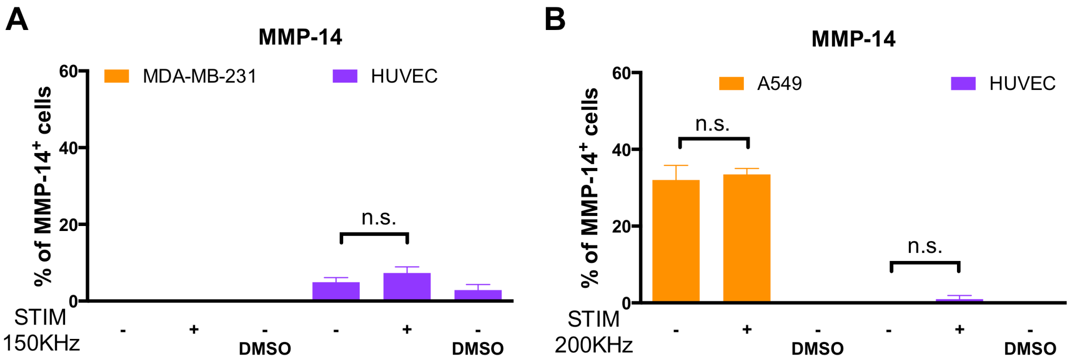

Supplementary Figure S3. (A) Percentage of MMP-14^{+} cancer cells (MDA-MB-231) and MMP-14^{+} endothelial cells (HUVEC) stimulated at a frequency of 150 kHz and intensity of 1.1 V/cm. (B) Percentage of MMP-14^{+} in lung cancer cells (A549) and endothelial cells (HUVEC) stimulated at a frequency of 200 kHz and intensity of 1.1 V/cm. Unpaired t-test, n.s=not significant.

Supplementary references

1 Mark, J.E. Polymer data handbook. Oxford University Press: *New York* (1999).

2 Pavesi, A. *et al.* Electrical conditioning of adipose-derived stem cells in a multi-chamber culture platform. *Biotechnol. Bioeng.* **111** 1452-1463 (2014).

3 Sirivisoot, S., Pareta, R. & Harrison, B. S. Protocol and cell responses in three-

dimensional conductive collagen gel scaffolds with conductive polymer nanofibres for tissue regeneration. *Interface Focus* **4,** 20130050; doi:10.1098/rsfs.2013.0050 (2014).

4 Larmagnac, A., Eggenberger, S., Janossy, H. & Vörös, J. Stretchable electronics based on Ag-PDMS composites. *Sci Rep* **4** 7254. doi: 10.1038/srep07254 (2014).
